# A Happy New Year

**Published:** 1915-01

**Authors:** 


### A Happy New Year.

It is somewhat difficult to anticipate, incongruous to offer delayed Holiday sentiments. We may be pardoned, therefore, for alluding in retrospect to three Holidays recently past. Except for the European war, and on that account, only by contrast, was it possible for our people to celebrate Thanksgiving in a spirit of worldly sincerity. Yet, with the realization that matters generally were about as bad as they could be short of the horrors of war, our country had reason to be thankful for an abundance of most of the necessities of life and for the existence of recent legislation and a disposition to modify business methods so as to make real wealth rather than its tokens, the gauge of prosperity.

We trust that Christmas has been sanely observed, without waste of time or money on unneeded and unwanted "remembrances,'' without fostering precocious and false standards among the children of the well-to-do, without neglect of the happiness of the children of the poor. More particularly, we hope that the Christmas waste of a few years ago has been made effective in stimulating business and has been applied to the real suffering of our near neighbors across the Atlantic. We hope also that the seals betokening a worthy philanthropy have not marred anyone's pleasure by reminding them of bacteria, morbidity and lingering death.

By the time that this issue reaches our readers, the waste baskets and the bath room will have been cleared of decorated tissue paper and the prosaic modern substitute for the old Twelfth Night festivities will have reduced the household to its normal state of order. The New Year brings with it new responsibilities and new hopes based on opportunities. What is needed is not so much new resolutions of personal or national good conduct as a general effort, partly legitimately selfish, mainly altruistic, to secure a ready and economic exchange of material wealth and honest, efficient labor, and, if need be, independently of abnormal conditions affecting the artificial medium of exchange—money. Supplies, hoarded because unsalable; mental, muscular and mechanic power, idle because of lack of business and non-employment are different phases of the same economic disease.

